# Supplementary material for: Machine Learning Accelerates Crystallization for Structure Determination
Source: Angew Chem Int Ed Engl. 2026 May 4;65(25):e1218503. doi: 10.1002/anie.1218503 (PMC13266958; doi:10.1002/anie.1218503)

checkCIF/PLATON report

Structure factors have been supplied for datablock(s) lcz015

THIS REPORT IS FOR GUIDANCE ONLY. IF USED AS PART OF A REVIEW PROCEDURE FOR PUBLICATION, IT SHOULD NOT REPLACE THE EXPERTISE OF AN EXPERIENCED CRYSTALLOGRAPHIC REFEREE.

No syntax errors found.   CIF dictionary   Interpreting this report

Datablock: lcz015

Bond precision:	C-C = 0.0191 A		Wavelength=1.54184
Cell:	a=10.2262(2)	b=17.2521(4)	c=21.9165(5)
	alpha=83.212(2)	beta=84.130(2)	gamma=87.082(2)
Temperature:	100 K		

	Calculated	Reported
Volume	3816.40(15)	3816.40(15)
Space group	P -1	P -1
Hall group	-P 1	-P 1
Moiety formula	C23 H11 Ag3 F18 N6 O2, C15 H3 Ag3 F18 N6, 3(C8 H8 O2)	2(C15 H3 Ag3 F18 N6), 4(C8 H8 O2)
Sum formula	C62 H38 Ag6 F36 N12 O8	C62 H38 Ag6 F36 N12 O8
Mr	2410.27	2410.26
Dx,g cm-3	2.098	2.097
Z	2	2
Mu (mm-1)	13.480	13.480
F000	2328.0	2328.0
F000’	2338.98	
h,k,lmax	13,21,27	12,21,27
Nref	16396	15759
Tmin,Tmax	0.199,0.198	0.511,1.000
Tmin’	0.088	

Correction method= # Reported T Limits: Tmin=0.511 Tmax=1.000
AbsCorr = MULTI-SCAN

```
Data completeness= 0.961          Theta(max)= 78.623

R(reflections)= 0.0919( 11691)                     wR2(reflections)=
                                                    0.2175( 15759)
S = 1.049                    Npar= 1125
```

The following ALERTS were generated. Each ALERT has the format
test-name_ALERT_alert-type_alert-level.
Click on the hyperlinks for more details of the test.

Alert level B

```
PLAT780_ALERT_1_B Coordinates do not Form a Properly Connected Set     Please Do !
```

Author Response: The alert is due to a large number of co-crystallized molecules in the which sometimes do not show as a connected set. This is acceptable from a crystallographic point of view.

Alert level C

```
PLAT042_ALERT_1_C Calc. and Reported MoietyFormula Strings  Differ     Please Check
              Calc: C23 H11 Ag3 F18 N6 O2, C15 H3 Ag3 F18 N6, 3(C8
                     H8 O2)
              Rep.: 2(C15 H3 Ag3 F18 N6), 4(C8 H8 O2)
PLAT213_ALERT_2_C Atom F00H           has ADP max/min Ratio .....        3.2 prolat
PLAT213_ALERT_2_C Atom F019           has ADP max/min Ratio .....        3.2 prolat
PLAT213_ALERT_2_C Atom F01K           has ADP max/min Ratio .....        3.4 prolat
PLAT213_ALERT_2_C Atom F01V           has ADP max/min Ratio .....        3.6 prolat
PLAT213_ALERT_2_C Atom F01X           has ADP max/min Ratio .....        3.8 prolat
PLAT221_ALERT_2_C Solv./Anion  Resd 2  F   Ueq(max)/Ueq(min) Range       4.2 Ratio
PLAT234_ALERT_4_C Large Hirshfeld Difference N01J     --C02U     .      0.17 Ang.
PLAT234_ALERT_4_C Large Hirshfeld Difference C02R     --C030     .      0.16 Ang.
PLAT342_ALERT_3_C Low Bond Precision on  C-C Bonds ..............    0.01914 Ang.
PLAT415_ALERT_2_C Short Inter D-H..H-X       H00R     ..H027     .      2.03 Ang.
                                                  1+x,y,z  =      1_655 Check
PLAT906_ALERT_3_C Large K Value in the Analysis of Variance ......    22.002 Check
PLAT906_ALERT_3_C Large K Value in the Analysis of Variance ......     4.375 Check
PLAT906_ALERT_3_C Large K Value in the Analysis of Variance ......     2.432 Check
PLAT911_ALERT_3_C Missing FCF Refl Between Thmin & STh/L=    0.600        80 Report
               -9 13  0,   8-13  1,   9-13  1,   9-12  1,   9-11  1,   9-10  1,
               -9  5  1,  -9 12  1,  -8 14  1,   9-10  3,   9 -8  3,  11  9  3,
               -6 15  4,  -6 16  4,  -7-16  5,  -6 16  5,  -9  0  7,  -4  1  7,
               -7  0  9,   0 20  9,   4 -8 10,   1  5 10,   1  2 12,  -7 13 12,
                4 17 12,   1 19 12,  -5 14 13,  -4 15 13,  -1 16 13,   1 19 13,
                   (   50 More Missing: see the .ckf listing file)
PLAT971_ALERT_2_C Check Calcd Resid. Dens.  1.16Ang From Ag02          2.24 eA-3
PLAT971_ALERT_2_C Check Calcd Resid. Dens.  1.19Ang From Ag01          1.91 eA-3
PLAT971_ALERT_2_C Check Calcd Resid. Dens.  0.95Ang From Ag03          1.91 eA-3
PLAT971_ALERT_2_C Check Calcd Resid. Dens.  1.01Ang From Ag05          1.89 eA-3
PLAT971_ALERT_2_C Check Calcd Resid. Dens.  1.04Ang From N01J          1.85 eA-3
PLAT971_ALERT_2_C Check Calcd Resid. Dens.  1.02Ang From Ag03          1.85 eA-3
```

PLAT971_ALERT_2_C Check Calcd Resid. Dens. 1.11Ang From Ag06 1.81 eA-3
PLAT971_ALERT_2_C Check Calcd Resid. Dens. 1.00Ang From N00U 1.77 eA-3
PLAT971_ALERT_2_C Check Calcd Resid. Dens. 1.24Ang From Ag06 1.76 eA-3
PLAT971_ALERT_2_C Check Calcd Resid. Dens. 1.03Ang From Ag01 1.76 eA-3
PLAT971_ALERT_2_C Check Calcd Resid. Dens. 1.17Ang From Ag04 1.74 eA-3
PLAT971_ALERT_2_C Check Calcd Resid. Dens. 1.23Ang From Ag03 1.71 eA-3
PLAT971_ALERT_2_C Check Calcd Resid. Dens. 1.14Ang From Ag05 1.57 eA-3
PLAT971_ALERT_2_C Check Calcd Resid. Dens. 1.10Ang From Ag01 1.51 eA-3
PLAT972_ALERT_2_C Check Calcd Resid. Dens. 0.75Ang From Ag06 -1.78 eA-3
PLAT972_ALERT_2_C Check Calcd Resid. Dens. 0.69Ang From Ag03 -1.74 eA-3
PLAT972_ALERT_2_C Check Calcd Resid. Dens. 0.64Ang From Ag01 -1.65 eA-3
PLAT972_ALERT_2_C Check Calcd Resid. Dens. 0.75Ang From Ag03 -1.60 eA-3
PLAT972_ALERT_2_C Check Calcd Resid. Dens. 0.86Ang From Ag05 -1.54 eA-3
PLAT972_ALERT_2_C Check Calcd Resid. Dens. 0.94Ang From Ag04 -1.51 eA-3
PLAT975_ALERT_2_C Check Calcd Resid. Dens. 0.85Ang From O00R . 1.45 eA-3
PLAT977_ALERT_2_C Check Negative Difference Density on H00R . -0.38 eA-3
PLAT977_ALERT_2_C Check Negative Difference Density on H01R . -0.36 eA-3

Alert level G

PLAT003_ALERT_2_G Number of Uiso or U(i,j) Restrained non-H-Atoms 14 Report
PLAT004_ALERT_5_G Polymeric Structure Found with Maximum Dimension 1 Info
PLAT007_ALERT_5_G Number of Unrefined Donor-H Atoms .... 4 Report
H00K H00F H00J H00R
PLAT083_ALERT_2_G SHELXL Second Parameter in WGHT Unusually Large 195.39 Why ?
PLAT154_ALERT_1_G The s.u.'s on the Cell Angles are Equal ..(Note) 0.002 Degree
PLAT177_ALERT_4_G The CIF-Embedded .res File Contains DELU Records 2 Report
PLAT178_ALERT_4_G The CIF-Embedded .res File Contains SIMU Records 1 Report
PLAT186_ALERT_4_G The CIF-Embedded .res File Contains ISOR Records 5 Report
PLAT188_ALERT_3_G A Non-default SIMU Restraint Value has been used 0.0200 Report
PLAT242_ALERT_2_G Low 'MainMol' Ueq as Compared to Neighbors of C02K Check
PLAT242_ALERT_2_G Low 'MainMol' Ueq as Compared to Neighbors of C02N Check
PLAT242_ALERT_2_G Low 'MainMol' Ueq as Compared to Neighbors of C038 Check
PLAT242_ALERT_2_G Low 'MainMol' Ueq as Compared to Neighbors of C03E Check
PLAT242_ALERT_2_G Low 'MainMol' Ueq as Compared to Neighbors of C03F Check
PLAT242_ALERT_2_G Low 'MainMol' Ueq as Compared to Neighbors of C03G Check
PLAT242_ALERT_2_G Low 'MainMol' Ueq as Compared to Neighbors of C01O Check
PLAT242_ALERT_2_G Low 'MainMol' Ueq as Compared to Neighbors of C02I Check
PLAT242_ALERT_2_G Low 'MainMol' Ueq as Compared to Neighbors of C02M Check
PLAT242_ALERT_2_G Low 'MainMol' Ueq as Compared to Neighbors of C02R Check
PLAT242_ALERT_2_G Low 'MainMol' Ueq as Compared to Neighbors of C035 Check
PLAT242_ALERT_2_G Low 'MainMol' Ueq as Compared to Neighbors of C03C Check
PLAT380_ALERT_4_G Incorrectly? Oriented X(sp2)-Methyl Moiety ..... C02W Check
PLAT720_ALERT_4_G Number of Unusual/Non-Standard Labels .... 162 Note

Ag01	Ag02	Ag03	Ag04	Ag05	Ag06	F007	F008
F009	F00A	F00B	F00C	F00D	F00E	O00F	H00F
F00G	F00H	O00I	O00J	H00J	O00K	H00K	O00L
F00M	F00N	F00O	N00P	F00Q	O00R	H00R	F00S
N00T	N00U	F00V	N00W	F00X	F00Y	F00Z	N010
O011	F012	N013	N014	F015	F016	F017	N018
F019	F01A	F01B	F01C	F01D	C01E	N01F	O01G
N01H	C01I	H01I	N01J	F01K	F01L	N01M	C01N
H01N	C01O	C01P	F01Q	C01R	H01R	C01S	H01S
C01T	C01U	H01U	F01V	C01W	H01W	F01X	F01Y
C01Z	H01Z	C020	C021	C022	C023	H023	C024
C025	C026	H026	C027	H027	C028	C029	H029
C02A	H02A	C02B	C02C	C02D	H02I	C02E	H02J

```
              C02F    C02G    H02G    C02H    C02I    C02J    C02K    F02L
              C02M    C02N    C02O    C02P    H02P    C02Q    H02Q    C02R
              C02S    C02T    C02U    C02V    H02V    C02W    H02E    H02F
              H02H    C02X    H02K    H02L    H02M    C02Y    H02Y    C02Z
              H02B    H02C    H02D    C030    C031    H031    C032    H032
              C033    C034    C035    C036    H03A    H03C    H03D    C037
              C038    C039    C03A    C03B    H03B    C03C    F03D    C03E
              C03F    C03G
PLAT802_ALERT_4_G CIF Input Record(s) with more than 80 Characters          1 Info
PLAT860_ALERT_3_G Number of Least-Squares Restraints ............          94 Note
PLAT883_ALERT_1_G Absent Datum for _atom_sites_solution_primary ..     Please Do !
PLAT910_ALERT_3_G Missing FCF Reflection(s) Below Theta(Min)[Deg]=       3.10 Note
                0  1  0,   0  0  1,
PLAT912_ALERT_4_G Missing # of FCF Reflections Above STh/L=  0.600        553 Note
PLAT933_ALERT_2_G Number of HKL-OMIT Records in Embedded .res File         27 Note
               -9  5  1,  -9  5 16,  -9 12  1,  -9 13  0,  -8  7 17,  -8 10 16,
               -8 11 15,  -8 14  1,  -7  8 18,  -6  6 19,  -5 14 13,  -4 14 14,
               -4 15 13,  -1  4 24,  -1  6 23,  -1  8 22,  -1 20 13,   0  6 23,
                1  2 23,   2  4 21,   8-13  1,   9-13  1,   9-12  1,   9-11  1,
                9-10  1,   9-10  3,   9 -8  3,
PLAT941_ALERT_3_G Average HKL Measurement Multiplicity ..........         2.9 Low
PLAT969_ALERT_5_G The 'Henn et al.' R-Factor-gap value ..........       2.856 Note
              Predicted wR2: Based on SigI**2  7.62 or SHELX Weight 20.73
PLAT978_ALERT_2_G Number C-C Bonds with Positive Residual Density.          0 Info
```

```
   0 ALERT level A = Most likely a serious problem - resolve or explain
   1 ALERT level B = A potentially serious problem, consider carefully
  38 ALERT level C = Check. Ensure it is not caused by an omission or oversight
  32 ALERT level G = General information/check it is not something unexpected

   4 ALERT type 1 CIF construction/syntax error, inconsistent or missing data
  46 ALERT type 2 Indicator that the structure model may be wrong or deficient
   9 ALERT type 3 Indicator that the structure quality may be low
   9 ALERT type 4 Improvement, methodology, query or suggestion
   3 ALERT type 5 Informative message, check
```

Validation response form

Please find below a validation response form (VRF) that can be filled in and pasted into your CIF.

```
# start Validation Reply Form
_vrf_PLAT042_lcz015
;
PROBLEM: Calc. and Reported MoietyFormula Strings  Differ     Please Check
RESPONSE: ...
;
_vrf_PLAT213_lcz015
;
PROBLEM: Atom F00H            has ADP max/min Ratio .....        3.2 prolat
RESPONSE: ...
;
_vrf_PLAT221_lcz015
;
PROBLEM: Solv./Anion  Resd 2  F   Ueq(max)/Ueq(min) Range        4.2 Ratio
RESPONSE: ...
```

```
;
_vrf_PLAT234_lcz015
;
PROBLEM: Large Hirshfeld Difference N01J     --C02U     .       0.17 Ang.
RESPONSE: ...
;
_vrf_PLAT342_lcz015
;
PROBLEM: Low Bond Precision on  C-C Bonds ...............    0.01914 Ang.
RESPONSE: ...
;
_vrf_PLAT415_lcz015
;
PROBLEM: Short Inter D-H..H-X       H00R     ..H027     .       2.03 Ang.
RESPONSE: ...
;
_vrf_PLAT906_lcz015
;
PROBLEM: Large K Value in the Analysis of Variance ......     22.002 Check
RESPONSE: ...
;
_vrf_PLAT911_lcz015
;
PROBLEM: Missing FCF Refl Between Thmin & STh/L=    0.600         80 Report
RESPONSE: ...
;
_vrf_PLAT971_lcz015
;
PROBLEM: Check Calcd Resid. Dens.  1.16Ang From Ag02            2.24 eA-3
RESPONSE: ...
;
_vrf_PLAT972_lcz015
;
PROBLEM: Check Calcd Resid. Dens.  0.75Ang From Ag06           -1.78 eA-3
RESPONSE: ...
;
_vrf_PLAT975_lcz015
;
PROBLEM: Check Calcd Resid. Dens.  0.85Ang From O00R    .       1.45 eA-3
RESPONSE: ...
;
_vrf_PLAT977_lcz015
;
PROBLEM: Check Negative Difference Density on H00R      .      -0.38 eA-3
RESPONSE: ...
;
# end Validation Reply Form
```

It is advisable to attempt to resolve as many as possible of the alerts in all categories. Often the minor alerts point to easily fixed oversights, errors and omissions in your CIF or refinement strategy, so attention to these fine details can be worthwhile. It is up to the individual to critically assess their own results and, if necessary, seek expert advice.

PLATON version of 17/09/2025; check.def file version of 17/09/2025

duplicate check

No duplication found

Datablock lcz015 - ellipsoid plot

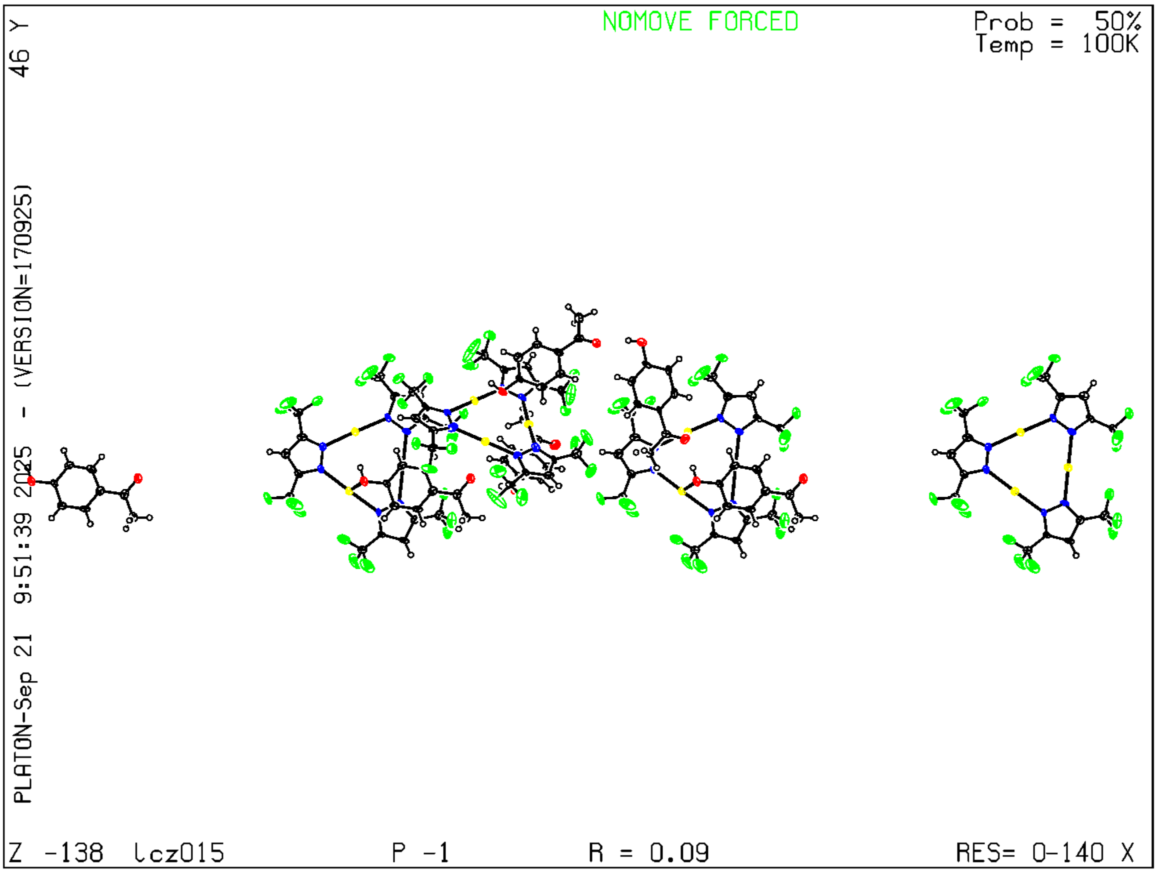

Supplement: Supplementary file 2 — Supporting File 2: anie72462‐sup‐0002‐DataFile.zip. [file ANIE-65-e1218503-s002.zip › supplementary_materials_crystallographic_data submit.zip!/cif_and_checkcif(1-114)/Ag3Pz3_15/Ag3Pz3_15_checkcif.pdf]
